# MRI-Determined Psoas Muscle Fat Infiltration Correlates with Severity of Weight Loss during Cancer Cachexia

**DOI:** 10.3390/cancers13174433

**Published:** 2021-09-02

**Authors:** Lisa Patzelt, Daniela Junker, Jan Syväri, Egon Burian, Mingming Wu, Olga Prokopchuk, Ulrich Nitsche, Marcus R. Makowski, Rickmer F. Braren, Stephan Herzig, Mauricio Berriel Diaz, Dimitrios C. Karampinos

**Affiliations:** 1 Department of Diagnostic and Interventional Radiology, Klinikum Rechts der Isar, Technical University of Munich, 81675 Munich, Germany; daniela.junker@tum.de (D.J.); jan.syvaeri@tum.de (J.S.); egon.burian@tum.de (E.B.); mingming.wu@tum.de (M.W.); marcus.makowski@tum.de (M.R.M.); rbraren@tum.de (R.F.B.); dimitrios.karampinos@tum.de (D.C.K.); 2 Department of Surgery, Klinikum Rechts der Isar, Technical University of Munich, 81675 Munich, Germany; olga.prokopchuk@tum.de (O.P.); ulrich.nitsche@tum.de (U.N.); 3 Institute for Diabetes and Cancer, Helmholtz Center Munich, 85764 Neuherberg, Germany; stephan.herzig@helmholtz-muenchen.de (S.H.); mauricio.berrieldiaz@helmholtz-muenchen.de (M.B.D.); 4 Joint Heidelberg-IDC Translational Diabetes Program, Inner Medicine 1, Heidelberg University Hospital, 69120 Heidelberg, Germany; 5 Chair Molecular Metabolic Control, Technical University Munich, 81675 Munich, Germany; 6 Deutsches Zentrum für Diabetesforschung, 85764 Neuherberg, Germany

**Keywords:** magnetic resonance imaging, cancer cachexia, weight loss, skeletal muscle, proton density fat fraction (PDFF)

## Abstract

**Simple Summary:**

During cancer cachexia, patients experience profound weight loss due to metabolic dysfunction, resulting in muscle composition alterations. The proton density fat fraction (PDFF) using magnetic resonance imaging (MRI) is an established biomarker to assess changes of muscle composition. We evaluated the suitability of MRI-determined psoas and erector spinae muscle PDFF and fat volume as biomarkers for monitoring cachexia development in an oncological cohort of 58 patients and assessed the regional variation in muscle parameters over time. Our results indicate that psoas muscle PDFF and fat volume could serve as MRI-determined biomarkers for early risk stratification and disease monitoring regarding progression and severity of weight loss in cancer cachexia.

**Abstract:**

Purpose: To evaluate the suitability of psoas and erector spinae muscle proton density fat fraction (PDFF) and fat volume as biomarkers for monitoring cachexia severity in an oncological cohort, and to evaluate regional variances in muscle parameters over time. Methods: In this prospective study, 58 oncological patients were examined by a 3 T MRI receiving between one and five scans. Muscle volume and PDFF were measured, segmentation masks were divided into proximal, middle and distal muscle section. Results: A regional variation of fat distribution in erector spinae muscle at baseline was found (*p* < 0.01). During follow-ups significant relative change of muscle parameters was observed. Relative maximum change of erector spinae muscle showed a significant regional variation. Correlation testing with age as a covariate revealed significant correlations for baseline psoas fat volume (r = −0.55, *p* < 0.01) and baseline psoas PDFF (r = −0.52, *p* = 0.02) with maximum BMI change during the course of the disease. Conclusion: In erector spinae muscles, a regional variation of fat distribution at baseline and relative maximum change of muscle parameters was observed. Our results indicate that psoas muscle PDFF and fat volume could serve as MRI-determined biomarkers for early risk stratification and disease monitoring regarding progression and severity of weight loss in cancer cachexia.

## 1. Introduction

Cachexia is a complex syndrome characterized by weight loss due to depletion of muscle and fat mass [1]. Also known as wasting disease, cachexia is associated with underlying illness such as cancer, heart failure and chronic obstructive pulmonary disease. It affects up to 23% of all cancer patients and can be accounted for up to 30% of all cancer deaths [2,3,4,5]. Patients with cancer of the gastrointestinal tract, especially patients with tumors of the esophagus, stomach, colon and pancreas, often develop cachexia: 40% to 83% of patients with these cancer entities develop cachexia during the course of the disease [6,7]. A formal definition of cachexia was proposed by Fearon et al. in an international consensus defining cachexia as the unintentional loss of more than 5% body weight within 6 months or the weight loss of more than 2% while BMI < 20 kg/m^2^ or sarcopenia (index of skeletal muscle of extremities for men lower than 7 × 26 kg/m^2^ and for women lower than 5 × 45 kg/m^2^) with any weight loss >2% body weight [8].

Nearly 60% of all neoplastic diseases occur in patients older than 65 years [9]. Dietary deficiencies are especially common and hazardous for elderly cancer patients. In this patient group, cachexia is associated with higher mortality, reduced quality of life, decreased tolerance and response to therapy and diminished capability to recover [10,11]. Therefore, a valid monitoring method for quantitative cachexia risk stratification is necessary.

Loss of skeletal muscle is a key feature in patients with cachexia [12] and is therefore a potential target for monitoring cachexia progression and for cachexia risk stratification. As the exact pathophysiology of cachexia is yet to be understood, the weight loss is thought to be associated with cytokine excess, indicating a central role of a chronic inflammatory response in disease etiology [13,14]. Furthermore, increased muscle protein breakdown and decreased muscle protein synthesis can be observed [14]. Skeletal muscle contains contractile, i.e., functional tissue, and ectopic fat, which can be divided into intermuscular and intramuscular fat. Intermuscular fat is placed beneath the fascia and between muscle strands, while intramuscular fat is found within muscle fibers. Intermuscular fat content progresses with age and is associated with obesity [15,16]. Recently, it was shown that ectopic fat is not evenly distributed along the length of the muscle but that the proximal and distal muscle sections present higher fat fractions than the middle section of the muscle both in healthy subjects and those suffering from Duchenne muscular dystrophy [17]. A heterogenous fat allocation along the vertical and horizontal muscle axis has been also observed in cancer patients [18].

With regard to the tremendous impact of cancer cachexia on treatment outcome, survival and quality of life of cancer patients, non-invasive methods for an early detection of cachexia and further disease monitoring are needed in order to allow for early intervention in cases of prospective disease progression. To date, cachexia is mainly monitored with regular weight controls and evaluation of lost skeletal muscle mass through measurements of extremity circumference or ultrasound measurements of muscle thickness. However, in patients with advanced cancer, the intra- and extracellular water content can be increased due to tumor mass blocking lymphatic fluid drainage paths, certain drugs and altered metabolism, resulting in edema and ascites [19,20]. This may result in overestimated weight and circumferences, leading to a false cachexia progression estimation. Instead, many studies on cancer cachexia used computed tomography (CT) to examine changes in body composition [21,22], as CT is part of the clinical staging routine and is therefore easy to implement for monitoring tissue changes. CT allows us to determine muscle volume and muscle fat infiltration by measuring the Hounsfield Unit (HU) of individual muscles. It does not provide measurements of volumetric changes of contractile muscle volume or muscle fat volume. Consequently, the precise composition of skeletal muscle cannot be deducted from CT images and thus, little is known of how the composition of skeletal muscle evolves during the course of cancer. Moreover, CT is accompanied by high radiation exposure and is therefore not suitable for examinations outside the clinical routine.

In contrast to CT, MRI is radiation-free and provides the possibility to directly measure muscle fat content and thus differentiate between muscle fat and contractile tissue. Furthermore, MR imaging can provide spatially resolved 3D information, unlike dual-energy x-ray absorptiometry (DXA), which is also often used for body composition analysis. The most frequently used MRI methods to study muscle composition are chemical shift encoding-based fat quantification techniques [23,24]. After consideration of multiple confounding factors, these techniques measure the proton density fat fraction (PDFF), which is defined as the proportion of mobile protons attributable to fat in relation to all mobile protons, including water protons [25,26]. PDFF mapping allows spatially resolved fat quantification in different tissues, for example in liver and adipose tissue, and thus has become a popular metabolic phenotyping parameter [27].

Therefore, the aim of this study was to (1) investigate the temporal course of changes in muscle composition as a potential cachexia biomarker, using MRI-based PDFF with regard to both intra- and intermuscular fat; (2) examine the regional variation in muscle composition and location-dependent predisposition to cachexia-induced muscle changes; and (3) evaluate the role of initial PDFF and fat volume distribution on prospective BMI changes in a cohort with underlying neoplastic diseases.

## 2. Materials and Methods

### 2.1. Study Design and Subjects

Between August 2017 and March 2020, a total of 58 patients newly diagnosed with cancer (21 female, 37 male) were recruited via consultations at the Department of Surgery and Internal Medicine at Klinikum Rechts der Isar, Munich, and in collaboration with the Roman-Herzog-Cancer-Center, Munich. Study protocols and procedures were approved by the ethical committee of the Faculty of Medicine of the Technical University of Munich, Germany. Prior to scanning, written informed consent was obtained from all patients.

Cancer entities included ductal adenocarcinoma of the pancreas, adeno- and squamous cell carcinoma of the esophagus, stomach, colon or rectum, bronchial carcinoma and squamous cell carcinoma of the oropharynx. Exclusion criteria were pregnancy and standard MRI contraindications. Therapy regimes experienced by subjects included surgical therapy, radiotherapy and chemotherapy. Thirty seven patients received chemotherapy, 27 as neoadjuvant therapy, 9 adjuvant after surgery and 6 as palliative care. Seven patients received neoadjuvant radiotherapy during the course of the study.

To monitor changes during the course of the disease, baseline and follow-up MRI scans were scheduled. Patients were asked to participate in the short follow-up MRI scans whenever a clinical follow-up examination was scheduled. Overall, 89 study scans were performed of which 32 longitudinal scans were completed on 22 patients. Of those, all 22 patients received one follow-up, seven patients received two follow-ups, two patients received three follow-ups and one patient received four follow-ups. Time intervals between baseline and first follow-up varied between 44 and 239 days, mean time from baseline to the first follow-up was 123 days. Of the 36 patients who did not receive follow ups, one patient started their therapy regimen at another site and was not available, two patients’ general health status did not allow further MRI scans, eight patients died before a follow-up could be scheduled and 25 were unavailable for unknown reasons. During the course of this study 11 patients died.

### 2.2. Anthropometric Measurements

Weight was assessed in light clothing before each MRI scan using MPD 250K100M (Kern and Sohn, 72336 Balingen, Germany). Weight loss prior to the baseline scan was self-reported. Height was self-reported. BMI was calculated as the quotient of weight in kilogram and height in meters squared (kg/m^2^). Circumferences of waist (at height of umbilicus) and right mid-thigh (halfway between knee and hip) were measured prior to each scan with a medical tape measure (KaWe Medizintechnik, 71679 Asperg, Germany).

### 2.3. MRI Measurements

MRI examinations of the abdomen and pelvis were performed on a 3T scanner (Elition; Philips Healthcare, Best, Netherlands). Subjects were placed in supine position and a combination of anterior and posterior coil arrays was used. Total scan time was reduced to a minimum (total scan time of 5 to 7 min) and embedded in the patient’s regular clinical follow-up schedule.

To measure PDFF and volume of skeletal muscle, a six-echo multi-echo gradient echo sequence with bipolar gradients was used. Four stacks covered the abdomen and pelvis from the liver dome to the femoral heads. Sequence parameters are shown in Table 1. Complex-based water–fat separation was performed. Specifically, PDFF maps were generated using the vendor’s online complex-based water–fat quantification algorithm, accounting for known confounding factors including the presence of multiple fat peaks, a single T_2_* correction and eddy-current-induced phase errors.

### 2.4. Skeletal Muscle Segmentation

**Figure 1:**
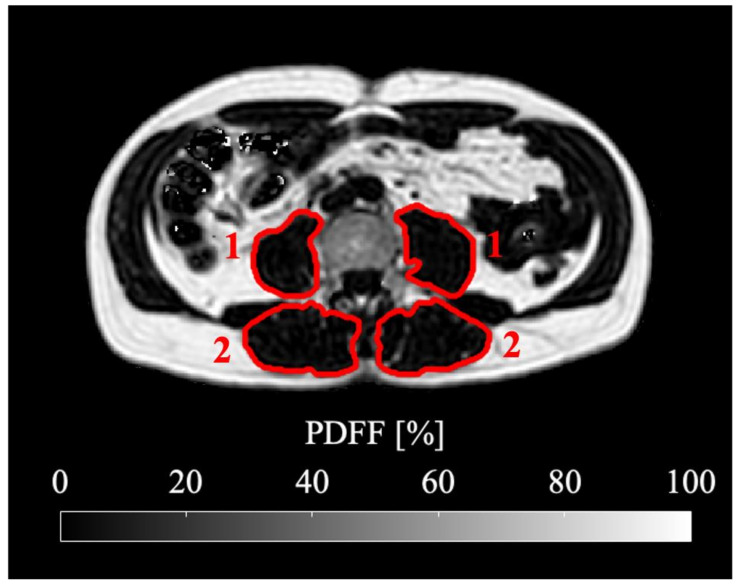
Representative segmentation of psoas (1) and erector spinae (2) muscle shown in the PDFF map at the level of the fourth lumbar vertebra (L4). The subject is a 60-year-old male with a BMI of 26.0 kg/m^2^ suffering from ductal adenocarcinoma of the pancreas.

Manual segmentation was performed on the water-separated images merged over the four stacks. Psoas and erector spinae muscle were segmented using the polygon-tool in the free open-source software ITK-SNAP [28] (www.itksnap.org, accessed on 3 February 2020). Reliability of the manual segmentation was ensured by defining the target area precisely and reviewing the segmentation with a board certified radiologist (7 years of experience in whole-body imaging). Psoas muscle was segmented bilaterally from its origin to the myotendinous junction at the height of the acetabulum. Erector spinae muscle included M. iliocostalis, Mm. intertransversarii, Mm. levatores costarum, Mm. interspinales, Mm. rotatores breves and longi, M. multifidus and M. semispinalis. Erector spinae muscle was segmented bilaterally from the height of the liver dome to its most caudal insertion at the Os sacrum. A representative muscle segmentation is displayed in Figure 1. In order to exclude unintentionally included bony structures, an automated post processing step removed voxels with T_2_* values below 5 ms and above 100 ms from the PDFF analysis (Python version 2.7; https://www.python.org/, accessed on 6 April 2020).

In two patients, no muscle segmentations could be performed due to metal-induced artifacts and a missing sequence, respectively. Both patients were excluded from the final analysis. In one patient, only psoas segmentation could be performed due to metal induced artifacts within the erector. This means a total of 88 psoas segmentations and 86 erector spinae segmentations were analyzed.

Total muscle volume and average PDFF (in %) were extracted for both psoas and erector muscles. Contractile tissue volume was calculated as $total\;muscle\;volume-\left(PDFF\times total\;muscle\;volume\right).$ Muscle fat volume was calculated as PDFF×total muscle volume.

To conduct a regional analysis of the described muscle parameters, a custom-built Python algorithm was used. Thereby, the segmentation masks of the whole muscle were lengthwise divided into equal thirds, resulting in individual masks for the proximal, middle and distal muscle section.

### 2.5. Statistical Analysis

The collected parameters were normally distributed according to the Kolmogorov–Smirnov test; thus, data are expressed as mean ± standard deviation (SD) (range in parentheses), if not otherwise denoted. In case the data showed log-normal distributions, logarithmic transformation was performed, which was true for erector PDFF, erector spinae total muscle volume and fat volume as well as for psoas PDFF. Differences between the baseline measurements in cachectic vs. non-cachectic patients were tested by means of the independent samples *t*-test. Statistical significance of observed maximum absolute and relative change in muscle parameters was tested with the one-sample *t*-test (test value = 0). Differences in absolute and relative maximum change of psoas and erector spinae muscle parameters per muscle segment were tested with one-way ANOVA analysis. Correlation analyses were performed using Pearson correlation, with logarithmic transformation if appropriate. Spearman’s Rank correlation coefficient was used when one or both parameters did not show normal distribution after logarithmic transformation. Partial correlation with age as control variable was conducted. Statistical analysis was performed by using IBM SPSS Statistics for Macintosh (Version 26.0., IBM Corp. Released 2019, Armonk, NY, USA). A two-sided *p* value of 0.05 was considered statistically significant and no correction was made for multiple testing because of the explorative character of the study.

## 3. Results

### 3.1. Characteristics of the Study Cohort

In the analyzed 56 patients, mean BMI was 25.4 ± 4.4 kg/m^2^ (range: 13.8 kg/m^2^ to 38.4 kg/m^2^). Mean age was 63.0 ± 11.8 years (range: 37 to 83 years). Mean age in women was 62.7 ± 12.7 years (range: 40 to 83 years), in men it was 63.1 ± 11.1 years (range: 37 to 78 years). On average, patients reported to have lost 4.3 ± 5.5 kg (range: −20 to 0 kg) body weight prior to their baseline examination. Based on this information, 22 patients were cachectic at the time of baseline, of which 7 received at least one follow-up scan. During the course of this study, 11 patients, who were not cachectic at baseline, became cachectic according to the definition by Fearon et al. [8]

Skeletal muscle measurements at baseline for psoas and erector spinae muscle are shown in Table 2. Mean PDFF at baseline was 9.7% in psoas muscle and 15.5% in erector spinae muscle. Regional analysis revealed unequal distribution of fat in both psoas and erector spinae muscles in proximal, middle and distal muscle sections at baseline. The observed mean psoas PDFF was higher in the proximal muscle section than in the middle and distal muscle section (*p* = 0.26, *p* = 0.05).

Mean erector spinae PDFF was significantly higher in the distal muscle section than in the proximal and middle muscle section (*p* < 0.01). As anticipated, the middle section of psoas and erector spinae muscle presented with a significantly larger muscle volume, contractile tissue volume and fat volume than the proximal and distal muscle section (*p* < 0.01).

No significant difference could be found in the measured total muscle parameters at baseline for the cachectic vs. non-cachectic patient groups, neither with the self-reported weight loss, nor with the measured weight loss or both combined.

### 3.2. Longitudinal Analysis

**Figure 2:**
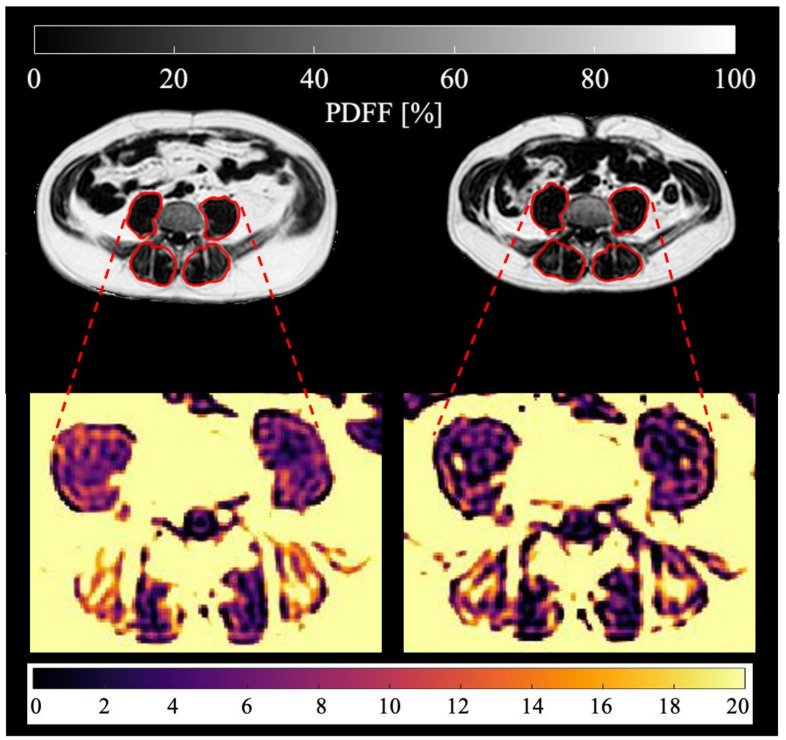
Representative PDFF maps of the psoas and erector spinae muscles (marked in red) of the same patient (male, 56 years old, BMI at baseline 28.6 kg/m^2^, BMI at follow-up 24.2 kg/m^2^, suffering from adenocarcinoma of the esophagogastric junction) are shown. Baseline scan is on the left, third follow-up scan after 218 days on the right. Mean psoas PDFF at baseline: 12.02%, mean erector spinae PDFF at baseline: 16.69%, mean psoas PDFF at follow-up: 8.62%, mean erector spinae PDFF at follow-up: 14.85%.

**Figure 3:**
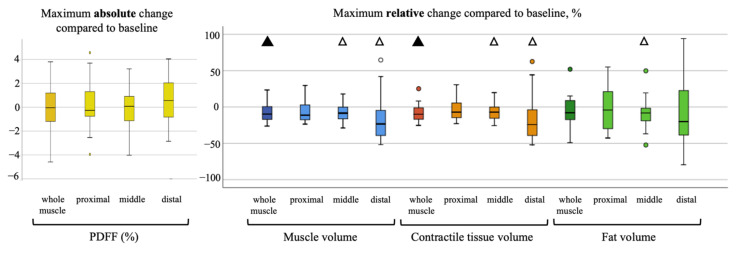
Visualization of absolute maximum change of M. psoas PDFF on the left, relative maximum change of muscle volume, contractile tissue volume and fat volume per proximal, middle and distal muscle section on the right. Statistical significance of change tested with one-sample *t*-test (compared to no change with test value = 0), △= <0.05, ▲= <0.01. No statistical significance of change differing between muscle sections (tested with one-way ANOVA analysis) was found.

**Figure 4:**
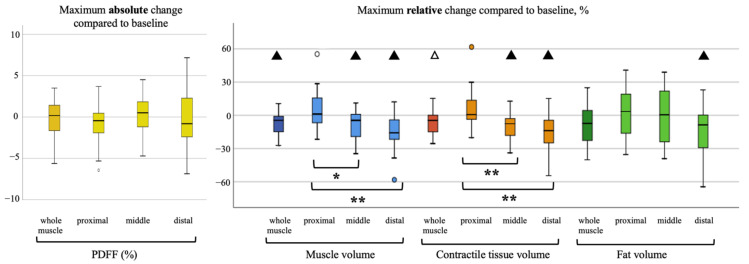
Visualization of absolute maximum change of M. erector spinae PDFF on the left, relative maximum change of muscle volume, contractile tissue volume and fat volume per proximal, middle and distal muscle section on the right. Statistical significance of change tested with one-sample *t*-test (compared to no change with test value = 0), **△**= <0.05, ▲= <0.01. Statistical significance of change differing between muscle sections tested with one-way ANOVA analysis, * = <0.05, ** = <0.01.

During the follow-ups, the patients lost on average BMI points of 2.11 kg/m^2^ ± 1.99, (range, −6.11–1.38). In the analyzed skeletal muscle, there was an average loss of total volume, fat volume and contractile tissue volume. A representative PDFF map of a longitudinal scan is shown in Figure 2. Maximum changes (i.e., in case of more than one follow-up, the maximum change in comparison to the baseline) of PDFF (absolute) and skeletal muscle parameters (relative) are visualized in Figure 3 and Figure 4. Maximum relative changes of erector spinae muscle volume and contractile tissue volume showed a significant spatial variation when performing regional analysis for a proximal, middle and distal muscle section. On average, both muscles lost muscle volume, contractile tissue volume and fat volume. A significant maximum relative change of muscle volume was observed for the whole psoas and erector spinae muscle (−9.2%, *p* < 0.01; −7.7%, *p* < 0.01). Both middle and distal muscle sections of psoas and erector spinae muscle exhibited significant relative maximum change of muscle volume (psoas: −7.6%, *p* = 0.01, −15.2%, *p* < 0.01; erector spinae: −8.1%, *p* < 0.01, −15.0%, *p* < 0.01). On average, a significant relative maximum change of contractile tissue volume for the whole psoas and erector spinae muscle was observed (−8.9%, *p* < 0.01; −6.2% *p* = 0.02). The middle and distal muscle sections of both muscles underwent a significant relative maximum change of contractile tissue (psoas: −7.4%, *p* = 0.01, −15.5%, *p* = 0.03; erector spinae −9.0%, *p* < 0.01, −14.8%, *p* < 0.01). A significant relative maximum change in fat volume was observed in the psoas middle muscle section (−9.1%, *p* < 0.05) and erector spinae distal muscle section (−14.3%, *p* < 0.01).

No statistically significant absolute change in PDFF for psoas and erector spinae muscle was observed in this study.

### 3.3. Correlation Analysis

At baseline, age correlated with PDFF of both the psoas and the erector spinae (r = 0.53, *p* < 0.01 and r = 0.56, *p* < 0.01, respectively). Furthermore, age correlated with erector spinae fat volume (r = 0.4, *p* < 0.01) and inversely with psoas total and contractile volume (r = −0.36, *p* < 0.01 and r = −0.39, *p* < 0.01, respectively). At baseline, BMI also correlated with erector spinae total muscle, fat and contractile volume (r = 0.7, r = 0.65 and r = 0.57, all *p* < 0.01) and with psoas total muscle, fat and contractile volume (r = 0.35, *p* = 0.01; r = 0.6, *p* < 0.01 and r = 0.31, *p* = 0.02, respectively).

**Figure 5:**
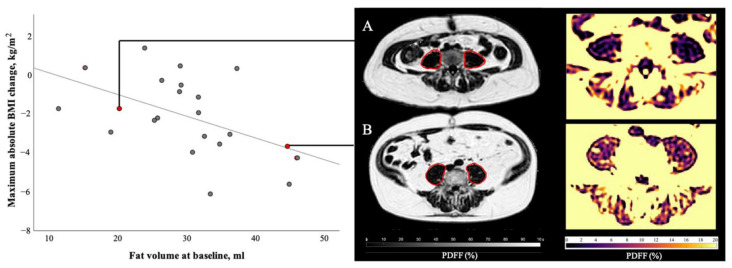
Correlation of psoas muscle fat volume at baseline and maximum absolute BMI change during the course of disease. At baseline patient A (female, 48 years old, BMI 26.2 kg/m^2^, suffering from colorectal cancer) presented with 20.2 mL of psoas muscle fat volume, patient B (male, 64 years old, BMI 33.1 kg/m^2^, suffering from ductal adenocarcinoma of the pancreas) with 44.6 mL of psoas muscle fat volume. During the course of this study, patient A lost 1.7 BMI points and patient B lost 3.7 BMI points. Statistical analysis of all 22 patients with follow-ups revealed a significant correlation (r = −0.51, *p* = 0.02) between baseline psoas fat volume and maximum BMI change during follow-ups.

The correlation analysis of baseline muscle parameters and maximum absolute BMI change at follow-ups is described in Table 3. Importantly, when correlating the baseline measurements of skeletal muscle with the maximum changes in BMI throughout the follow-ups, an inverse correlation between the maximum BMI change and the baseline psoas fat volume could be found (r = −0.51, *p* = 0.02; Figure 5). Bivariate correlation with age as a control variable enhanced this correlation (r = −0.55, *p* < 0.01) and furthermore lead to inverse correlations between the maximum BMI change and the baseline psoas PDFF (r = −0.52, *p* = 0.02), as well as the erector spinae fat volume (r = −0.46, *p* = 0.04).

Correlating the baseline measurements of skeletal muscle per muscle section with the maximum changes in BMI revealed an inverse correlation between BMI change and psoas baseline muscle parameters of the distal muscle section. The muscle volume, contractile tissue volume and fat volume of the distal muscle section showed significant correlation with maximum BMI change (r = −0.44, *p* = 0.04, r = −0.44, *p* = 0.04, r = −0.56, *p* < 0.01). Psoas fat volume of the proximal muscle section also correlated with maximum BMI change (r = −0.44, *p* = 0.04), and partial correlation with age as a control variable improved this correlation (r = −0.53, *p* = 0.01). When correlation analysis of erector spinae muscle baseline parameters was conducted, a correlation between maximum BMI change and muscle volume of middle and distal muscle section (r = −0.43, *p* = 0.05, r = −0.44, *p* = 0.04), contractile tissue volume of the distal section (r = −0.46, *p* = 0.03) and fat volume of the proximal section (r = −0.49, *p* = 0.02) was found. Adding sex as a covariate instead of age did not lead to any significant correlations between the maximum BMI change and the skeletal muscle measurements.

## 4. Discussion

During the course of the present study, subjects lost on average BMI, total muscle volume as well as fat and contractile tissue volume in both psoas and erector spinae muscle. Even though muscle PDFF did not exhibit significant changes during follow-ups, psoas and erector spinae muscle were subjected to profound alterations, as the presented analysis of total and regional muscle volume, contractile tissue volume and fat volume revealed.

At baseline, BMI correlated with total muscle, fat and contractile volume of both psoas and erector spinae muscle. Strikingly, psoas fat volume at baseline correlated inversely with maximum BMI change during the follow-ups. Baseline psoas PDFF also correlated inversely with maximum BMI change when age was added as a control variable. As we now demonstrated using MRI, the loss of total and fat volume in muscle during cancer cachexia has been shown by others for different tumor entities using computed tomography before [21,29,30]. These studies also found a correlation between muscle loss and low muscle attenuation and overall survival. Studies on advanced cancer patients utilizing DXA techniques showed a loss of body fat and muscle mass during the course of the disease [31,32]. Even though a high correlation of DXA-derived appendicular lean tissue mass to both MRI- and CT-derived analysis of skeletal muscle mass is known [33,34], it was shown that DXA overestimates whole body lean tissue mass compared to skeletal muscle measurements with MRI [34,35]. Skeletal muscle fat infiltration, also known as myosteatosis, cannot be assessed with DXA techniques [36,37]. Moreover, when using DXA, edema, dehydration or any changes in the patient’s fluid status prevent exact analysis of lean soft tissue mass and composition [38,39]. While CT and DXA are commonly used for body tissue quantification, volumetric changes of contractile muscle volume and muscle fat volume can only be identified with MR imaging, which is not accompanied by harmful radiation, unlike CT and DXA. In the present study a chemical shift encoding-based fat quantification technique was used. The robust technique of proton density fat fraction mapping was utilized for spatially resolved fat quantification.

### 4.1. Regional Analysis of Psoas and Erector Spinae Muscle

At baseline, psoas and erector spinae muscle displayed unequal fat distribution along the proximodistal axis. Follow-ups revealed a significantly different maximum relative change of muscle volume, contractile tissue volume and fat volume in the different muscle sections of the erector spinae muscle. Thereby, middle and distal muscle sections experienced the largest change. Precise fat distribution patterns of healthy and diseased muscle are unclear and regional analysis of muscle undergoing change due to cachexia has not been conducted before. A regional variation of fat infiltration pattens has been previously reported for other pathological conditions; for example, Duchenne muscular dystrophy, which is accompanied with higher fat fractions in the proximal and distal muscle regions [17]. Local differences in muscle composition need to be considered when planning clinical muscle biopsies. This regional variation has to be studied more intensively in order to gain an understanding of muscular atrophy and the underlying pathology.

### 4.2. Muscle PDFF as a Predictive Marker

At baseline, BMI correlated with total muscle, fat and contractile tissue volume of both psoas and erector spinae. Age, which was shown to correlate with baseline PDFF in psoas and erector spinae in this study, seems to be a stronger driver of increasing fat deposition in paraspinal muscles than BMI in women [40]. The present results suggest that a high muscle PDFF of psoas depending on the patient´s age and psoas fat volume at baseline is associated with greater weight loss during the course of the disease. Therefore, muscle PDFF and the calculated intramuscular fat volume could be predictive markers for risk stratification regarding the development and severity of cancer cachexia.

The importance of psoas muscle as a marker for cachexia-induced whole-body changes is unclear, as the literature remains indecisive. It has been previously proposed that psoas muscle cross-sectional area could be a potential marker for progression of cachexia and is associated with complications and adverse outcomes [41,42]. Other studies using the muscle cross sectional area found that psoas muscle alone might not be a reliable marker for cachexia-induced changes in body composition [43,44].

Therefore, in the present study, two paraspinal muscles, psoas and erector spinae muscle, were segmented. Examining both psoas and erector spinae muscles allows us to further examine the impact of cachexia on both intra- and intermuscular fat. Whereas psoas muscle presents as one continuous muscle strand with a muscle belly at the widest diameter, the erector spinae muscle consists of different muscle groups. Fatty infiltration of psoas muscle, as measured in this study, is mainly influenced by changes in intramuscular fat. Erector spinae muscle, on the other hand, commonly demonstrates fatty streaks in between the muscle groups, thereby exhibiting a high occurrence of intermuscular fat. Our results indicate that erector spinae total muscle parameters, and therefore whole muscle intermuscular fat infiltration, do not correlate with cachexia progression and severity. On the contrary, psoas muscle PDFF as a marker for intramuscular fat and fat volume has shown significant association with occurrence of greater weight loss during cancer cachexia.

Sarcopenia, the loss of skeletal muscle mass and muscle function, was shown to affect overweight patients especially negatively. Sarcopenic obesity was found to be associated with adverse outcomes in cancer patients [45,46]. Skeletal muscle mass depletion in an overweight body cannot be easily assessed by BMI calculations or circumference measurements. MR imaging is a suitable method to evaluate muscle mass and composition in overweight cancer patients [47].

### 4.3. Clinical Implications

As cachexia is a progressive disease and is assumed to be susceptible to therapy only in the early stages, timely intervention is favorable [8]. Common therapy approaches focus on improved nutrition, as it was shown that an increase in energy intake is associated with increased survival [48]. Moreover, therapy with anti-inflammatory drugs, like cyclo-oxygenase or cytokine inhibitors, has been shown to positively affect the patient [49,50]. However, no widely accepted anti-cachexia therapy regimen has been implemented yet. Since numerous novel drugs and different lifestyle interventions are currently being developed and tested, reliable cachexia monitoring parameters need to be established [51]. Early cachexia diagnostics in the clinical setting is necessary to administer therapy options in time. By implementing methods to evaluate the anticipated risk of severe cachexia development, patients at high risk can be identified and given intensified anti-cachexia therapy including improved nutrition, lifestyle interventions or anti-inflammatory drugs. The findings of the presented study seek to contribute to developing cachexia diagnostic tools that are able to predict the patient’s individual risk of developing severe cancer cachexia in the future.

### 4.4. Limitations

When interpreting the presented data, certain limitations have to be considered.

First, the heterogeneity of the underlying cancer entities with consecutive differing chemotherapeutic regimes leads to different side effects. A known side effect of Platinum-based drugs often used for chemotherapy is cachexia, weight loss and muscle wasting [52,53]. However, sufficient research about the prevalence and exact impact of chemotherapy on muscle volume and fat infiltration is missing. Therefore, in this study, we did not take the effect of chemotherapy on muscle wasting into account. Second, the study cohort presented a broad range of BMI, limiting the comparability of measurements. Furthermore, seven patients included in the follow-ups had already suffered from cachexia at baseline, when considering the self-reported prior weight loss. Third, a known limitation to PDFF measurements using MR are partial volume effects, which cannot be excluded at the borders of the measured tissue structures in this study, as an average PDFF per voxel (3 × 3 × 6 mm^3^) was calculated. The present work used a low spatial resolution in the PDFF mapping to complete the imaging of four stacks of the abdomen in four short breath-hold scans. Lastly, only a relatively small number of follow-ups were performed, with considerable variance in time intervals between baseline and follow-ups.

## 5. Conclusions

Psoas and erector spinae muscle presented an unequal fat distribution and local variation in responsiveness to cancer cachexia-induced changes. Muscle volume, contractile tissue volume and fat volume of erector spinae muscle experienced significant locally distinct changes. Thereby, middle and distal muscle sections were subject to a larger change. The findings of the present study indicate that psoas muscle PDFF and fat volume could be potential predictive biomarkers for risk stratification regarding the progression and severity of cancer cachexia. As reliable methods to foresee and assess cancer cachexia are still missing today, the results of this study are promising and suggest benefits to the use of muscle PDFF and fat volume as biomarkers for future catabolic body composition changes. Our results also reinforce the role of MR imaging in identifying and monitoring metabolic disorders. However, taking the small number of longitudinal data and the complex metabolic changes during cancer disease into account, the presented results need to be interpreted carefully and further research is needed to confirm our findings.

## Figures and Tables

**Table 1:** MRI sequence parameters for six-echo multi-echo gradient echo acquisition. Scan parameters per stack, a total of four stacks were performed each scan. MRI: magnetic resonance imaging, TR: repetition time, TE: echo time, SENSE: sensitivity encoding for accelerated MRI.

TR	7 ms
TE1/ΔTE	TE1 = 1.14 ms/ΔTE = 0.8 ms
Flip angle	3°
Bandwidth	2342.5 Hz/pixel
Acquisition matrix size	116 × 155
Field of view (FOV)	350 × 467 × 102 mm^3^
Acquisition voxel size	3 × 3 × 6 mm^3^
Slices	17
SENSE reduction factor	2.2 × 1.2
Number of averages	1
Scan time	8 s

**Table 2:** Skeletal muscle measurements at baseline for total psoas and erector spinae muscle and for proximal, middle and distal muscle section. PDFF: proton density fat fraction, SD: standard deviation. * no SD due to logarithmic transformation.

Muscle Measurements	Muscle Section	M. Erector Spinae, *n* = 55	M. Psoas, *n* = 56
		Mean (SD, Range)	Mean (SD, Range)
PDFF, %	total muscle	15.5 (*, 7.1–39.7)	9.7 (*, 5.6–22.9)
	proximal	14.1 (6.6, 4.1–39.1)	11.2 (3.8, 5.4–21.9)
	middle	15.2 (6.8, 5.9–37.8)	10.0 (3.5, 5.3–22.9)
	distal	22.5 (7.9, 8.1–43.9)	9.5 (4.0, 3.6–27.7)
Muscle volume, mL	total muscle	804.7 (*, 394.0–1588.9)	294.1 (115.3, 108.0–601.5)
	proximal	214.0 (65.9, 93.9–371.5)	75.0 (28.6, 33.3–151.1)
	middle	418.5 (136.7, 189.3–799.7)	166.7 (63.4, 59.3–335.8)
	distal	212.7 (93.0, 71.6–565.2)	52.4 (41.7, 10.5–202.7)
Fat volume, mL	total muscle	124.7 (*, 58.9–583.3)	28.5 (11.1, 10.5–59.4)
	proximal	29.6 (17.4, 8.2–117.2)	8.1 (3.4, 2.5–19.0)
	middle	62.6 (41.0, 24.5–296.6)	15.8 (5.8, 5.1–30.6)
	distal	46.4 (25.7, 19.2–169.5)	4.7 (4.8, 1.1–32.0)
Contractile tissue volume, mL	total muscle	706.6 (243.4, 307.2–1454.5)	265.6 (108.9, 93.9–567.1)
	proximal	184.5 (60.9, 85.0–333.4)	66.9 (26.8, 29.6–138.4)
	middle	355.9 (121.8, 152.1–737.3)	151.0 (60.0, 52.0–309.7)
	distal	166.3 (79.1, 49.5–464.7)	47.7 (37.7, 9.3–171.7)

**Table 3:** Correlation of baseline total muscle and proximal, middle and distal muscle section muscle parameters and absolute maximum BMI change at follow-up. Correlations are based on Spearman´s rank correlation as variables did not present normal distribution. PDFF: proton density fat fraction, n.s.: non-significant. Bold numbers higlight statistically significant r and *p* values.

Muscle Measurements	Muscle Section	Correlation Analysis, r (p)			
		M. Erector Spinae, *n* = 21		M. Psoas, *n* = 22	
		Maximum absolute BMI change, kg/m^2^	Correlation with age as control variable	Maximum absolute BMI change, kg/m^2^	Correlation with age as control variable
**PDFF, %**	total muscle	−0.12 (0.59)	−0.31 (0.18)	−0.29 (0.19)	**−0.52 (0.02)**
	proximal	−0.29 (0.21)	−0.42 (0.07)	−0.30 (0.18)	**−0.44 (<0.05)**
	middle	−0.18 (0.44)	−0.31 (0.19)	−0.28 (0.21)	−0.40 (0.08)
	distal	0.08 (0.74)	−0.02 (0.93)	−0.24 (0.29)	−0.36 (0.11)
**Muscle volume, mL**	total muscle	−0.41 (0.06)	−0.39 (0.09)	−0.25 (0.27)	−0.21 (0.36)
	proximal	−0.11 (0.64)	−0.14 (0.54)	−0.23 (0.30)	−0.08 (0.72)
	middle	**−0.43 (<0.05)**	−0.43 (0.05)	−0.06 (0.78)	−0.04 (0.88)
	distal	**−0.44 (0.04)**	**−0.45 (0.04)**	**−0.44 (0.04)**	−0.36 (0.11)
**Fat volume, mL**	total muscle	−0.35 (0.12)	**−0.46 (0.04)**	**−0.52 (0.02)**	**−0.55 (0.01)**
	proximal	**−0.49 (0.02)**	**−0.57 (<0.01)**	**−0.44 (0.04)**	**−0.53 (0.01)**
	middle	−0.36 (0.10)	**−0.45 (0.04)**	−0.33 (0.13)	−0.36 (0.11)
	distal	−0.16 (0.48)	−0.24 (0.30)	**−0.56 (<0.01)**	−0.40 (0.07)
**Contractile tissue volume, mL**	total muscle	−0.34 (0.13)	−0.30 (0.20)	−0.22 (0.34)	−0.17 (0.46)
	proximal	−0.10 (0.67)	−0.04 (0.85)	−0.20 (0.38)	−0.04 (0.88)
	middle	−0.38 (0.08)	−0.35 (0.12)	−0.07 (0.75)	−0.01 (0.99)
	distal	**−0.46 (0.03)**	**−0.44 (<0.05)**	**−0.44 (0.04)**	−0.35 (0.12)

## Data Availability

The processed data presented in this study are available on request from the corresponding author. The original imaging data are not publicly available due to privacy reasons given the sensitivity of patient data.
